# Medical Reform

**Published:** 1842-08-06

**Authors:** 


### THE MEDICAL EXAMINER.

### PHILADELPHIA, AUGUST 6, 1842.

We observe that a surgeon of some distinction in Albany, New York, has been mulcted in a fine little more than nominal, for denouncing the practice of an empirical cancer doctor, who eradicates cancers by means of an ointment, of which the most active ingredient is arsenic: the other constituents were drawn from a very reluctant witness on the trial, and thus the wonder-working secret stands exposed before the world. If this exposure should limit the extent of its employment, it may tend to benefit mankind, and prevent much unnecessary suffering; but, as usual in such cases, there is nothing in the nostrum to render it worthy of notice in a professional journal.

---

The agitation on the subject of medical reform appears to be unintermitting in England, and, judging from the correspondence of the London journals on the management of corporations and the administration of the poor laws, it seems that there is much need of amendment. The public estimate of professional standing in this country has been, and continues to be on the decline; but if there be relief in companionship under misfortune, we may feel ourselves in some degree consoled by the accounts from our father-land. It seems that, there as here, offices disgracefully mean in salary are caught at with avidity, and solemn compacts agreed upon at convocations of practitioners are almost immediately trampled upon by those who seemed eager for their enactment. The habit of prostration before the Ædile dignity of "committee men and trustees," for the smallest favour, appears to be even more marked in England than with us. The Colleges of Physicians and Surgeons are threatened, at present, with new constitutions at the hands of Government. We hope they will prove to be amended ones.

